# MiR-630 suppresses breast cancer progression by targeting metadherin

**DOI:** 10.18632/oncotarget.6339

**Published:** 2015-11-16

**Authors:** Ci-Xiang Zhou, Chen-Long Wang, An-Lu Yu, Qiu-Yu Wang, Meng-Na Zhan, Jun Tang, Xiu-Feng Gong, Qian-Qian Yin, Ming He, Jian-Rong He, Guo-Qiang Chen, Qian Zhao

**Affiliations:** ^1^ Department of Pathophysiology, Key Laboratory of Cell Differentiation and Apoptosis of National Ministry of Education, Shanghai Jiao Tong University School of Medicine (SJTU-SM), Shanghai 200025, China; ^2^ Institute of Health Sciences, SJTU-SM & Shanghai Institutes for Biological Sciences (SIBS), Chinese Academy of Sciences (CAS), Shanghai 200025, China; ^3^ Department of General Surgery, Rui-Jin Hospital, SJTU-SM, Shanghai 200025, China

**Keywords:** miR-630, MTDH, metastasis, breast cancer

## Abstract

MicroRNAs have been integrated into tumorigenic programs as either oncogenes or tumor suppressor genes. The miR-630 was reported to be deregulated and involved in tumor progression of several human malignancies. However, its expression regulation shows diversity in different kinds of cancers and its potential roles remain greatly elusive. Herein, we demonstrate that miR-630 is significantly suppressed in human breast cancer specimens, as well as in various breast cancer cell lines. In aggressive MDA-MB-231-luc and BT549 breast cancer cells, ectopic expression of miR-630 strongly inhibits cell motility and invasive capacity *in vitro*. Moreover, lentivirus delivered miR-630 bestows MDA-MB-231-luc cells with the ability to suppress cell colony formation *in vitro* and pulmonary metastasis *in vivo*. Further studies identify metadherin (MTDH) as a direct target gene of miR-630. Functional studies shows that MTDH contributes to miR-630-endowed effects including cell migration and invasion as well as colony formation *in vitro*. Taken together, these findings highlight an important role for miR-630 in the regulation of metastatic potential of breast cancer and suggest a potential application of miR-630 in breast cancer treatment.

## INTRODUCTION

Breast cancer remains to be the most common malignancy of women and causes 400,000 deaths annually worldwide [1]. With the advances in the early diagnosis and adjuvant treatment of breast carcinoma, the rate of five years disease-free survival in patients was increasing in recent decades. However, the majority of patients with metastatic disease will confront death between one and two years, even those treated with chemotherapy and caused tumor shrinkage at first [2]. Like that in other solid tumors, metastasis is the leading reason for the resultant mortality of patients with breast cancer [3, 4]. Tumor metastasis is the complex process which includes migratory tumor cells leaving the primary position through intravasation, disseminating throughout the body via the circulation, and eventually colonizing at distant organs [5]. Tumor cells acquire the ability of migration and invasion to depart from the original locality is the prerequisite of tumor metastasis [6, 7]. Although Epithelial–mesenchymal transition (EMT) is thought to be necessary for the progression of original tumor cells to metastatic cells or at least an alternative program, the causes which endue tumor cells with the migration and invasion capability are not fully understood. The mechanism underlying this lethal step of cancer process remains to be clearly illustrated [2].

MicroRNAs (miRNAs) are small, noncoding RNAs (18–23 nucleotides in size) that downregulate the expression of target genes by blocking translation or degradation of the mRNAs [8]. MiRNAs play important roles in various biological processes, including cell growth, differentiation, and development [9, 10]. As previously reported, more than 50% of miRNA-encoding loci reside in chromosomal altered regions [11], and expression profiling reveals that miRNAs were deregulated during tumorigenesis in various types of tumors [12]. In the last decade, an increasing number of miRNAs were identified as oncogenes or tumor suppressor genes [13–15]. Recent reports also indicate multiple functions of miRNAs in breast cancer metastasis. For examples, miR-373 and miR-520c promote invasion and metastasis of breast cancer cells by suppression of CD44 [16], while the miR-200 family members repress cell migration and invasiveness by targeting ZEB1 and ZEB2, known regulators of EMT [17, 18]. In our previous work, we showed that miR-124 inhibits invasive and metastatic potential of breast cancer by targeting the EMT regulator Slug [19].

MiR-630, identified from the miRNA cluster at chromosome 15q24.1, has been demonstrated to be deregulated and involved in several human malignancies. The present studies elucidate that its expression is decreased in various breast cancer cell lines as well as clinical breast cancer tissues, which might imply miR-630 as a tumor suppressor in breast cancer. Further studies indicate that miR-630 suppresses breast cancer cell migration, invasion and the ability of colony formation *in vitro* as well as lung metastasis *in vivo*. Metadherin (MTDH) that has been demonstrated a multifaceted oncogene which activates AKT, NFκB, and Wnt/β-catenin signal pathways is identified to be target gene of miR-630 and modulates the functions of miR-630 [20–24]. Herein, we report that miR-630 is significantly suppressed in human breast cancer specimens and cancer cell lines, and the miRNA suppresses breast cancer progression by targeting metadherin (MTDH).

## RESULTS

### MiR-630 is downregulated in the breast cancer clinical tissues and cell lines

**Figure 1:**
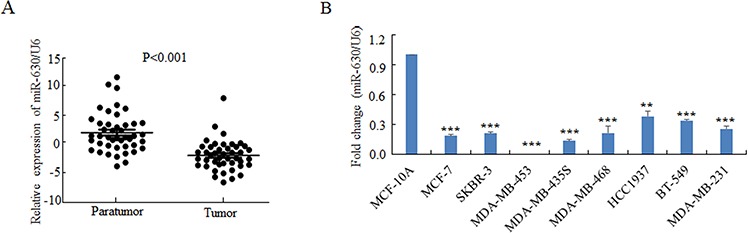
MiR-630 is downregulated in breast cancer tissues as well as breast cancer cell lines **A.** Quantitative PCR for comparing the expression levels of miR-630 in 43 paired clinical breast cancer cases. **B.** Quantitative PCR for detecting miR-630 expression levels in various breast cancer cell lines as indicted. Data represent mean ± SD of three independent experiments. **P < 0.001; **P < 0.001.

To investigate the roles of miR-630 in breast cancer progression, we detected the miR-630 expression levels by qRT-PCR between clinical breast carcinomas and paired adjacent non-neoplastic tissues from 43 cases of breast cancer patients. The characteristics of these clinical patients were shown in Supplementary Table S2. The result demonstrated that breast cancer tissues had lower miR-630 expression levels than the adjacent tissues in a statistically significant manner (*p* < 0.001) (Figure 1A). We also tested the expression of miR-630 by qRT-PCR in various breast cancer cell lines with a non-tumorigenic epithelial cell line MCF-10A as control. Consistently, the expression levels of miR-630 in all eight breast cancer cell lines tested were significantly reduced at different degrees compared to MCF10A cells (Figure 1B). To further validate that miR-630 expression levels decreased in the cancer tissues than the paired adjacent non-neoplastic tissues, the expression of miR-630 in validation cohort from 20 cases of breast cancer patients were measured. Supplementary Table S3 listed the characteristics of the validation cohort. The result showed that breast cancer tissues have lower miR-630 expression levels than the adjacent non-neoplastic tissues in a statistically significant manner (*p* < 0.001) (Supplementary Figure S1). Taken together, these results suggested that downregulation of miR-630 is a common event in breast cancer tissues, and thus it is inferred that miR-630 might involve in the pathogenesis of breast cancers.

### MiR-630 suppresses migration and invasion of breast cancer cells *in vitro*

**Figure 2:**
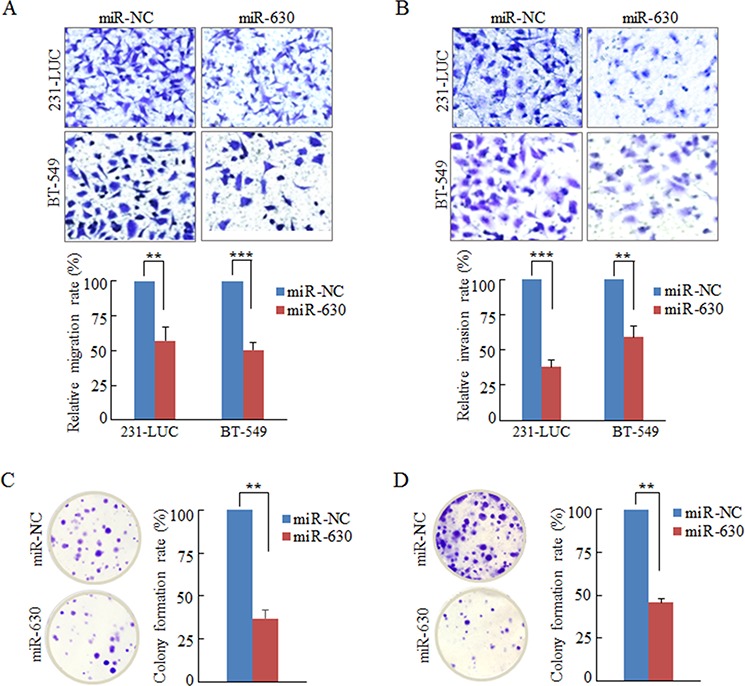
Ectopic expression of miR-630 suppresses Transwell migration, Matrigel invasion and colony formation in breast cancer cells *in vitro* **A.** Transwell migration assay measuring breast cancer cell migration in 231-LUC (top) and BT-549 (bottom) transiently transfected with miR-NC or mature miR-630 mimics, respectively. **B.** Matrigel invasion assay measuring breast cancer cell invasion in 231-LUC (top) and BT-549 (bottom) transfected with miR-NC or mature miR-630 mimics. Data represent mean ± SD of nine randomly selected areas from three independent experiments. **C, D.** Analysis of colony formation in 231-LUC cells (C) and BT-549 cells (D) transiently transfected with miR-NC or mature miR-630 mimics, respectively. **P < 0.01; ***P < 0.001. All experiments were repeated independent three times.

To evaluate the effect of miR-630 on breast cancer metastasis, the aggressive breast cancer cell lines MDA-MB-231-LUC cells (231-LUC) and BT-549 cells were transiently transfected with miR-630 mimics or non-specific miRNA (miR-NC), and the migration of these cells were measured by the Transwell migration assay. As shown in Figure 2A, miR-630 but not miR-NC dramatically suppressed the migratory property of these breast cancer cells. Wound healing assay also showed that miR-630 significantly inhibited the rate of wound closure of 231-LUC and BT-549 cells compared to control groups (Supplementary Figure S2A/S2B). Basement membrane is a specialized extracellular matrix (ECM) which plays vital roles in organizing epithelial tissues and is also the important barrier for tumor cells leaving the cradle [3, 26]. To mimic tumor cells traversing basement membrane, the matrigel invasion assay was performed as mentioned in Materials and Methods [27, 28]. Ectopic expression of miR-630 in 231-LUC and BT-549 cells strikingly reduced their invasion ability (Figure 2B). In addition, xCELLigence system with realtime technology allows us to observe the suppression of the migration and invasion ability of miR-630 in 231-LUC cells and BT-549 cells dynamically. The curves indicated that the disparity between miR-630 transfected cells and control groups were expanding with the extension of time (Supplementary Figure S2C/S2D).

### MiR-630 inhibits the colony formation ability of breast cancer cells *in vitro*

During the steps of invasion-metastasis cascade, cancer cells not only need to overcome at least three barriers (breaching basement membrane, intravasating into the blood vessels and extravagating into the parenchyma of distant tissues), but also have to survive in the circulation and reinitiate their proliferative programs at metastatic sites and generate macroscopic, clinically detectable neoplastic growths [26]. Hence, the colony formation assay was employed to assess the effect of miR-630 on cancer cell colony generation. The results revealed that miR-630 remarkably decreased the colony numbers of the breast cancer cell lines 231-LUC and BT-549 cells (Figure 2C/2D). Taken together, miR-630 inhibits the tumorigenesis in multi-steps including movement behavior, overcoming barriers and reinitiate their proliferative programs *in vitro*.

### MiR-630 directly targets MTDH in breast cancer cells and clinical tissues

**Figure 3:**
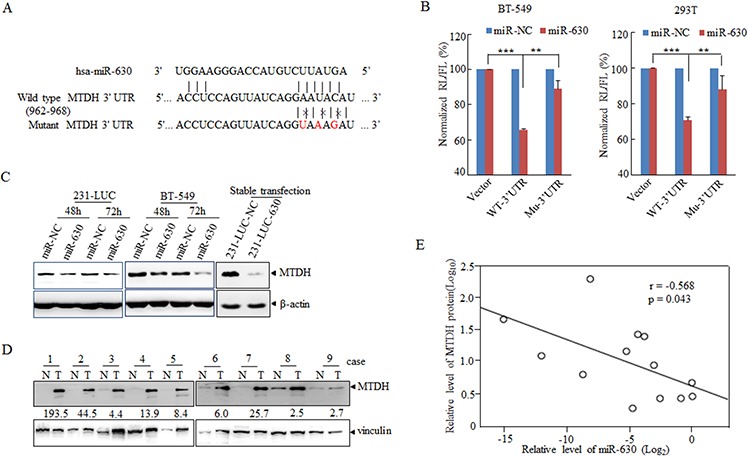
MiR-630 targets MTDH directly in breast cancer cell lines **A.** A schematic diagram illustrating the miR-630-binding sites and 3 mutated bases in MTDH 3′UTR. **B.** Dual-luciferase assays showing repression of wild-type UTR or mutant UTR following transfection of miR-630 or NC http://mimics.in BT-549 (Left) and HEK293T (Right) cells. Data represent mean ± SD. *P < 0.05; **P < 0.01; ***P < 0.001; All experiments were repeated independently three times. **C.** Western blot analysis showing the depression of MTDH in breast cancer cell lines 231-LUC (Left) and BT-549 (Right) transfected with miR-NC or miR-630 with β-actin as a loading control. **D.** Western blot describing the MTDH expression in clinical specimens with vinculin as a loading control. The folds change indicated the MTDH expression in tumors against paratumor normalized to vinculin. **E.** Expression and correlation of miR-630(Log_2_) and MTDH (log_10_) in paired clinical breast cancer samples.

To decipher the molecular mechanism of miR-630 mediates pathological functions in breast cancer metastasis, bioinformatics was applied broadly to predict the candidate targets of microRNAs [29]. At first, we analyzed the target genes by bioinformatics algorithms: TargetScan. Among these candidates, some oncogenes or migration associated genes including ARFGEF2, PDGFRA, SET as well as MTDH [30–33] were particularly concerned (Supplementary Figure S3). In order to validate whether these genes are the direct targets of miR-630, dual luciferase assay was performed. The wild-type 3′UTR (WT-3′UTR) of these genes were cloned into the downstream of the Renilla luciferase gene respectively in the psiCHECK vector with a firefly luciferase coding gene as internal control. HEK293T cells were transiently transfected with these constructs and miR-630 mimics or miR-NC. MiR-630 mimics rather than miR-NC suppressed the luciferase activity of reporter genes containing WT-3′UTR of these genes to some degree (Supplementary Figure S4A), whereas only the activity of 3′UTR of MTDH was reduced significantly in BT-549 breast cancer cells (Supplementary Figure S4B). In the further experiments, 3 bps mutation in the 3′UTR of MTDH gene corresponding to the putative miR-630 seed sequence (Figure 3A) had been cloned into the psiCHECK vector, the Renilla activity was refractory to suppression by miR-630 compared with WT-3′UTR of MTDH as observed in Figure 3B.

Next, we investigated the effect of miR-630 on suppression of MTDH 3′UTR. As for this, the expression levels of MTDH were measured in cells which were transfected transiently with miR-630 mimics or infected stably with lenti-viral carrying miR-630, and the result showed that MTDH was attenuated in both transient expression and stable expression cell lines compared to control groups (Figure 3C). In order to obtain more evidence, the expression levels of MTDH were detected in 13 paired human breast cancer samples selected randomly from the 43 paired samples which were used in Figure 1A, and it was markedly downregulated in tumor tissues compared with paired adjacent non-neoplastic tissues (Figure 3D). Moreover, the analysis of correlation of MTDH and miR-630 in these patient samples showed the inverse correlation between MTDH and miR-630 (Figure 3E). Overall, these results suggested MTDH was the potential functional target gene of miR-630.

### MTDH is involved in miR-630-regulated migration, invasion as well as colony formation

**Figure 4:**
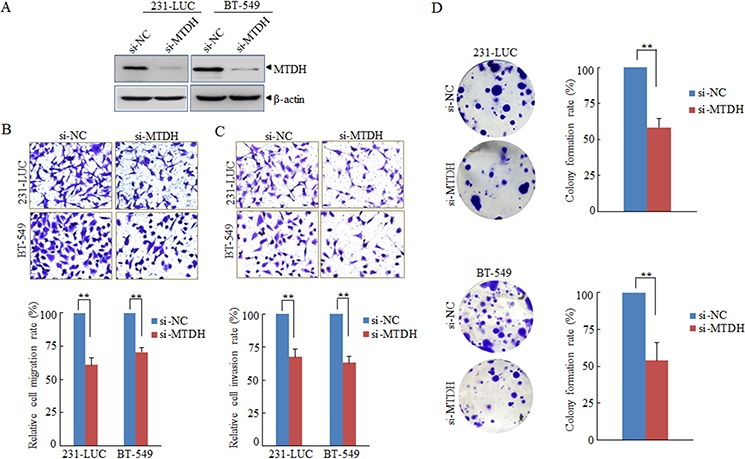
MTDH is involved in the pathological functions of miR-630 **A.** Western blot analyzing the efficiency of RNAi against MTHD with β-actin as a loading control. **B, C.** Effects of knockdown MTDH expression on Transwell migration (B) and Matrigel invasion (C) of 231-LUC and BT-549 transfected with si-NC or si-MTDH respectively. **D.** Effects of silencing MTDH expression on colony formation of 231-LUC cells (top) and BT-549 cells (bottom) transfected with si-NC or si-MTDH, respectively. Data represent mean ± SD. **P < 0.01. All experiments were repeated independent three.

To explore whether miR-630 exerts its function through its target gene MTDH. A loss-of-function assay was performed to validate pathological functions of miR-630 mediated by MTDH. As shown in Figure 4A, the expression of MTDH was downregulated by siRNA against MTDH both in 231-LUC and BT-549 cells. Colony formation assay, Transwell migration assay as well as matrigel invasion assay were performed, and the results revealed that suppression of MTDH could simulate the function of miR-630 that inhibit the property of colony formation, migration and invasion of breast cancer cells. (Figure 4B/4C/4D).

**Figure 5:**
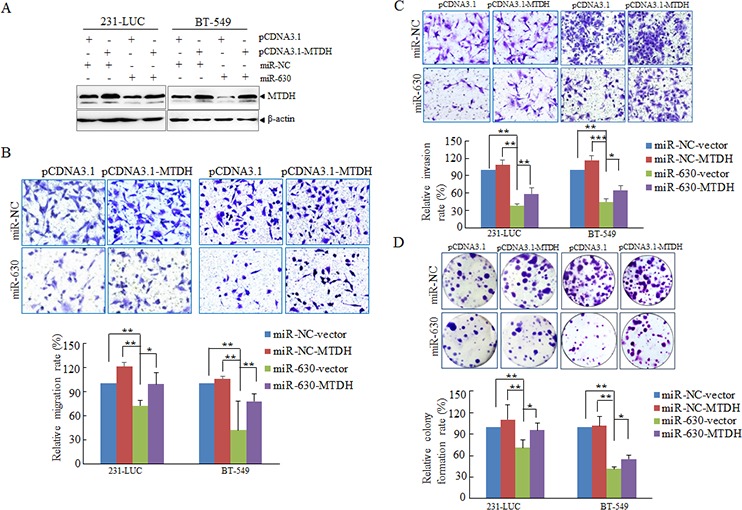
MTDH partially mediates pathology functions of miR-630 in breast cancer **A.** Western blot analyzing the expression of MTDH in 231-LUC cells and BT-549 cells transfected with miR-NC or miR-630 mimics together with either pcDNA3.1-MTDH or control vector with β-actin as a loading control. **B.** Transwell migration assay measuring migration of 231-LUC and BT-549 transfected with miR-NC or miR-630 mimics together with either pcDNA3.1-MTDH or control vector. **C.** Matrigel invasion assay measuring invasion of 231-LUC and BT-549 transfected with miR-NC or miR-630 mimics together with either pcDNA3.1-MTDH or control vector. **D.** Colony formation assay in 231-LUC cells and BT-549 cells transfected with miR-NC or miR-630 mimics together with either pcDNA3.1-MTDH or control vector. After transfection of pcDNA3.1-MTDH or control vector for 24 h, these cells were further transfected with miR-NC or miR-630 mimics. Data represent mean ± SD. *P < 0.05; **P < 0.01; ***P < 0.001. All experiments were repeated independent three times.

In order to further investigate the contribution of MTDH to the migration, invasion and colony formation, miR-NC or mature miR-630 was cotransfected with MTDH expression plasmid or its related vector respectively into breast cancer cells (Figure 5A). Next, Transwell migration assay matrigel invasion assay and colony formation assay were performed respectively, the pathological function of miR-630 were rescued partially as MTDH expression was restored to the original levels (Figure 5A/5B/5C/5D). The results further demonstrated that MTDH is involved in pathological actions of miR-630 in breast cancer metastasis.

### MiR-630 suppresses migration- metastasis cascade *in vivo*

To elucidate the *in vivo* effects of miR-630 on the breast cancer development, breast cancer-bearing mice have been applied. At first, two cell lines (231-LUC-miR-630 and 231-LUC-NC) had been generated. The expression levels of miR-630 were detected by q-PCR (Supplementary Figure S5A). Next, matrigel invasion assay and colony formation assay were performed to measure the effect of stable expression of miR-630 on 231-LUC cells, and the results showed that the property of colony formation and invasion of 231-LUC-630 was depressed significantly compared with 231-LUC-NC cells (Supplementary Figure S5B/S5C). At the same time, the proliferation of 231-LUC-NC cells and 231-LUC-630 cells were monitored, as the curve indicated, there is no difference between 231-LUC-630 cells and 231-LUC-NC cells, (Supplementary Figure S5D).

**Figure 6:**
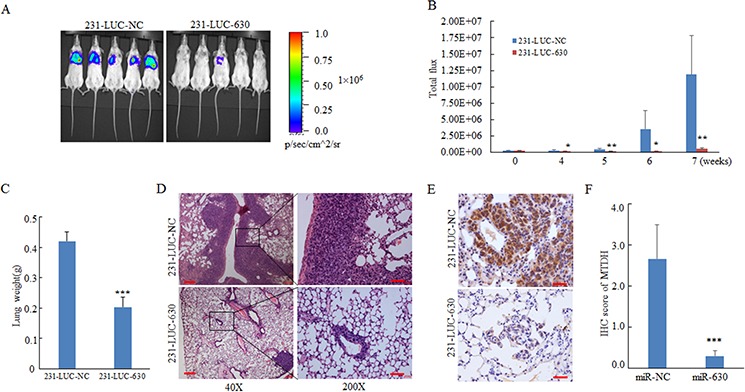
MiR-630 suppresses metastasis in a xenograft mouse model **A.** IVIS luciferase *in vivo* images of lung metastasis. **B.** Representative lung metastasis burden of xenografted animals on day 0, 14, 21, 28, 35, 42, 49 after injected with 231-LUC-NC cells or 231-LUC-miR-630 cells.; lung metastasis models were established (*n* = 6 per group) as described in “Materials and methods” and lung metastasis burden of xenografted animals were monitored using bioluminescent imaging (BLI). **C.** Lung tissue weight of the mice sacrificed on the day 49. **D, E.** Representative H&E staining (D) and MTDH immunohistochemical staining (E) of lung sections were performed on day 49 after cell injection. Scale bar, 50 μm. Lung tissues were fixed with 4% paraformaldehyde immediately after isolated for standard immunohistochemistry analysis as standard immunohistochemistry protocol. **F.** The IRS scores of MTDH expression in lung sections of mice orthotopically injected with 231-LUC-NC or 231-LUC-miR-630 cells. Data represent mean ± SD. *P < 0.05; **P < 0.01; ***P < 0.001.

In pulmonary metastasis model [34], 5 × 10^5^ cells were injected into NOD-SCID mice via tail veins. Lung metastasis burden of xenografted animals was detected using bioluminescent imaging (BLI). As shown in figure 6A, miR-630 repressed the formation of lung metastasis of 231-LUC cells. Moreover, lung metastasis of 231-LUC cells were monitored dynamically, and the results showed that the lung metastasis of 231-LUC-630 cells were impaired at the early stages of metastasis formation compared with 231-LUC-NC cells (Figure 6B). The lung metastasis can increase the weight of lung tissue. Accordingly, the weight of lung tissues from xenografted animals were measured. As expected, the increased gross weight of lung tissues was notably inhibited by miR-630 (Figure 6C). To further confirm the suppressive effect of miR-630 in tumor metastasis, histological analyses of lung tissues from mice were performed by using hematoxylin and eosin staining. The results suggested that lung tissues from mice injected with 231-LUC-630 cells showed small metastasis niduses, while lung tissues from NC group were heavily infiltrated (Figure 6D). To further determine whether MTDH mediates the pathologic functions of miR-630 *in vivo*, we assessed MTDH protein level in the lung sections of xenografted mice by immunohistochemical (IHC) staining. The mice lung sections presented different degrees of immunoreactive sores (IRS) were shown in Supplementary Figure S6. As the image indicated, the expression of MTDH was downregulated remarkably in miR-630 overexpressed group (Figure 6E/6F). Taken together, these results demonstrated that miR-630 showed multifunction in breast cancer metastasis processes including suppresses the ability of migration, invasion as well as cancer cell reinitiated proliferation in distant organ which were mediated by MTDH.

## DISCUSSION

Cancer metastasis accounts for more than 90% of all cancer related deaths [26, 35], blocking cancer metastasis would be an effective means for cancer treatment. In the present study, we have revealed that miR-630 is pathologically downregulated in various breast cancer cell lines and in the majority of human breast cancer tissues compared with the adjacent non-tumor tissue. Further functional analysis revealed the involvement of miR-630 in the progression of human breast cancer, and overexpression of miR-630 significantly decreased tumor lung metastasis and infiltration of breast cancer in the xenograft mouse model. Meanwhile ectopic overexpression of miR-630 significantly suppressed migration, invasion and colony formation in two aggressive breast cancer cell lines MDA-MB-231-luc and BT549 cells. Moreover, our study has identified MTDH, a multifaceted oncogene which activate AKT, NFκB, and Wnt/β-catenin signal pathways, as the direct target of miR-630 in breast cancer cells. These findings suggest that miR-630 plays an important role in the invasive and/or metastatic potential of breast cancer.

The expression of miR-630 is highly tissue and cancer type specific, thus demonstrating the functional and clinical significance of miR-630 may provide clinically relevant insights into its function and efficacious cancer management. For example, miR-630 expression in renal cancer specimens was remarkably higher than that in normal renal tissues, moreover, patients with higher expression of miR-630 in tumor tissue had a worse overall survival than patients with lower expression [36]. MiR-630 expression was significantly increased in hepatocellular carcinoma (HCC) tissues and cells compared with their normal counterparts which indicated that the elevation of miR-630 has an oncogenic role in the progression of HCC. However, recent studies show that miR-630 is downregulated in lung cancer and its expression inversely correlated with advanced-stage, higher grade lymph node status, invasion, poor overall survival, and poor disease free survival [37–40]. In this present study, the results indicated that the expression of miR-630 was downregulated in majority cases of 43 training cohort breast tumor specimens as well as 20 validation cohort breast tumor specimens (*P* < 0.001). Furthermore, correlation analysis revealed that downregulation of miR-630 has little correlation with clinicopathological traits including age, estrogen receptor, progesterone receptor, HER2, advanced-stage, as well as higher grade lymph node status (Supplementary Table S4). Taken together, these results might suggest that downregulation of miR-630 was a common event in breast cancer and miR-630 might participate in the multiple steps in the breast cancer developing process.

Besides the clinical relevance of miR-630 in different cancer tumors versus matched peritumors, miR-630 has also been reported to be involved in chemotherapy-related cell death. For example, miR-630 has been reported to regulate cisplatin-induced cell death in both non-small cell lung cancer as well as head and neck cancer, moreover, by targeting IGF-1R, miR-630 could induce apoptosis of pancreatic cancer cells. A previous study has also demonstrated miR-630 could improve patient response to HER-targeting drugs by targeting IGF-1R in HER2-overexpressing breast cancer. In the same study, the authors also showed that miR-630 was involved in cell motility and invasion in HER2-positive breast cancer cells [37]. Our functional study demonstrates that miR-630 significantly suppressed migration, invasion and colony formation in two aggressive breast cancer cell lines 231-luc and BT549 cells, which are negative of estrogen receptor (ER), progesterone receptor (PR) and HER2. It is well known that breast cancer is highly heterogeneous and can be divided into four major subtypes based on gene expression profiling: luminal A, luminal B, ErbB2, and basal-like. Basal-like breast cancer (BLBC) is characterized by the lack of expression of ER, PR and HER2. The absence of effective targeted therapies and poor response to standard chemotherapy often results in a rapidly fatal clinical outcome for BLBC. Our results indicated miR-630 play import roles in these aggressive breast cancer cells and provide a new clue in the treatment of BLBC.

Furthermore we identify a multifaceted oncogene, MTDH, as the direct target of miR-630 in breast cancer cells. Knock down of MTDH by siRNA could simulate the functions associated with miR-630; whereas, restoring the expression levels of MTDH in miR-630-overexpression cells could rescue the effects of miR-630. And more notably, staining MTDH in tumors derived from lung metastasis model that were employed in present work indicated that MTDH was downregulated remarkably in the miR-630 overexpressed group. Thus, MTDH has been identified as the mediator of pathological functions of miR-630 in breast cancer cells.

MTDH is located at chromosome region 8q22, a frequently amplified region in clinical breast cancers, promotes breast cancer development by enhancing chemoresistance and tumor cell adhesion to endothelial cells. Knockout mice model had documented that MTDH ubiquitously expresses in embryonic and adult organs of mouse, but MTDH is not essential for oncogenesis because that *Mtdh* KO mice were viable, fertile, and displayed no obvious abnormalities when monitored for up to 2 years. Surprisingly, MTDH is required for the expansion and function of both luminal and basal breast TICs in diverse breast tumor subtypes, but is non-essential for mammary stem cells, suggesting that it is different from many cell fate determinants that regulate both normal and cancerous stem cells [41]. In this study we revealed MTDH was the executor of pathology functional of miR-630, mediated the functions of miR-630 on suppressed migration and invasion or colony formation of breast cancer cells. These phenomenon suggest that the functional importance of MTDH-miR-630 makes it a potential therapeutic target in breast cancer treatment.

## MATERIALS AND METHODS

### Tissue specimens

Human Breast cancer and corresponding noncancerous tissues used in this study were obtained from patients who underwent surgical resection. This investigation included two independent patient groups, the training cohort and the validation cohort, the training cohort (*n* = 43) were collected from the Comprehensive Breast Health Center, Rui-Jin Hospital of Shanghai Jiao Tong University School of Medicine in 2011 and 2012. The validation cohort were collected from two hospitals: the First hospital Affiliated to Wenzhou Medical College (*n* = 8) and the Comprehensive Breast Health Center, Rui-Jin Hospital (*n* = 12). The samples were histologically confirmed by staining with hematoxylin and eosin. All experiments were performed under the approval of the Research Ethnics Committees of Shanghai Jiao Tong University School of Medicine and Wenzhou Medical College.

### Cell lines and cell culture

The MDA-MB-231-luc-D3H2LN (Xenogen, Alameda, CA) is a luciferase expressing cell line that was derived from parental MDA-MB-231 cells. This cell line has enhanced tumor take and metastatic potential compared to the parental MDA-MB-231. The MDA-MB-231-luc-D3H2LN (231-LUC) was propagated in Minimum essential medium with Earl's balanced salts solution (SH30024.01, Hyclone) medium supplemented with 10% fetal bovine serum (FBS, GIBCO, USA), 1% non-essential amino acids (30238.01, Hyclone) and 1% sodium pyruvate (30239.01, Hyclone). The MCF-10A cell, a non-tumorigenic epithelial cell line, was cultured using DMEM/F12 (11330, GIBCO) supplemented with 5% Horse serum (26050, GIBCO), 20 ng/ml EGF (AF-100–15, Peprotech), 0.5 mg/ml Hydrocortisone (07904, STEMCELL), 10 μg/ml Insulin (I5500, Sigma), 100 ng/ml Cholera toxin (c8052, Sigma), 1% Penicillin/Streptomycin (15140–122, GIBCO). Human breast cancer cell lines MDA-MB-231, MDA-MB-468 and MDA-MB-435S were kindly provided by Prof. Ming-Yao Liu (East China Normal University) were cultured in Leibovitz L-15 medium (11415–064, Gibco) with 10% fetal bovine serum (FBS). Cell lines BT-549, BT-474, SK-BR-3 and HCC1937 were cultured in RPMI 1640 (SH30027.01, Hyclone) with 10% FBS. The human breast cancer cell line MCF7 and the immortalized human embryonic kidney cell line HEK293T were cultured in Dulbecco's modified Eagle's medium with 10% FBS. All cell lines were fostered in a humidified atmosphere of 5% CO2 and 95% air except for MDA-MB-231, MDA-MB-468 and MDA-MB-435S, which were fostered at 37°C in a humidified atmosphere containing 100% air.

### RNA extraction and quantitative reverse transcription - PCR (q RT-PCR)

Total RNA was extracted from cells and tissues using TRIzol reagent (Invitrogen) following the manufacturer's protocol. The cDNA was synthesized according to the TaqMan MicroRNA Assay protocol with the gene-specific primers (ID:001563; ID: 001973). Real-time PCR was performed as TaqMan Small RNA Assays protocol on an ABI 7900HT fast real-time PCR system (Applied Biosystems). Expression data were uniformly normalized to the internal control U6 and the relative expression levels were evaluated using the 2^−ΔΔCt^ method.

### Plasmid construction and transfection

To validate the candidate target genes predicted by bioinformatics analysis are targets of miR-630, 3′UTRs of ARFGEF2, PDGFRA, SET, MTDH and EP300 containing the miR-630 binding site were amplified from the human genomic DNA using primers ARFGEF2-UTR-F/R, PDGFRA-UTR-F/R, SET-UTR-F/R, MTDH-UTR-F/R, severally (Supplementary Table S1), and then were cloned into the downstream of Renilla luciferase gene in the psiCHECK™-2 vector (Promega). Mutant MTDH-UTR plasmid containing three mutated bases on the predicted binding site was constructed using the fast mutagenesis kit (Vazyme) with primers MTDH-UTR-mut-F/R.

To stably overexpress mature miR-630 in MDA-231-D3H2LN cells, the DNA fraction containing the mature sequence of miR-630 was amplified with primers miR-630-F/R (Supplementary Table S1) from the model plasmid pcDNA^TM^6.2-GWSW/miR-630 kindly provided by Prof, Min-Liang Kuo (National Taiwan University) and then was cloned into the lentiviral expression plasmid pLVX-IRESZsGreen (Clontech Laboratories). The pcDNA3.1-MTDH plasmid was structured previously in our lab [25].

For co-expression of microRNA mimics and its target-genes, the target-gene MTDH was transfected using Hily Max (Dojindo, Kumamoto, Japan) firstly, and after 24 hours the microRNA mimics were transfected using Lipofectamine 2000 reagent (Invitrogen).

### RNA oligonucleotide

Both negative control (miR-NC) and miR-630 mimics were composed of RNA duplexes (GenePharma, Shanghai, China) with the following sequence orientated from 5′ to 3′, miR-630: AGUAUUCUGUACCAGGGAAGGU/CUUCCCUGGUACAGAAUACUUU and miR-NC: UU CUCCGAACGUGUCACGUTT/ACGUGACACGUUCG GAGAATT. The small interference RNAs siMTDH was double-strand RNAs consisted of the following sequence orientated from 5′ to 3: GGAGGAGGCUGG AAUGAAAdTdT /dTdTCCUCCUCCGACCUUACUUU (GenePharma, Shanghai, China). Neither microRNA mimics negative control nor small interfering RNAs negative control shares any homologous region with the human genome sequences.

### Cell proliferation and clonogenic assay

CCK-8 kit was used to analyze the changes of cell proliferation. The cells were embedded in 96-well plates (3 × 10^3^ cells per well) and incubated lasting 4 days at 37°C. The changes of cell proliferation was monitored every day, 10 μl of CCK-8 reagent (Dojindo, Kumamoto, Japan) was added into each well, and cells were incubated for another 1.5 hrs (BT-549) or 3 hrs (231-LUC) at 37°C. After gently shaken, the absorbance values were measured at 450 nm via a Hybrid Reader (BioTek Laboratory Instrument). To evaluate the clonogenic ability, cells were seeded in 6-well culture plates, continuously incubated for 14 days and the medium was changed every 3 days. The colonies were stained by crystal violet (1.5%, w/v; Sigma, St Louis, MO, USA).

### Transwell migration and matrigel invasion assay

Transwell migration and matrigel invasion assay were employed to determine the ability of cell migration and invasion using Transwell system (24-well, 8 μm pore size with polycarbonate membrane; Corning Costar, Lowell, MA, USA). For matrigel invasion assay, 4 hours before experiment, the polycarbonate membrane were plated by Matrigel (BD Biosciences, SanJose, CA, USA) diluted with serum-free medium, the ratio of Matrigel to serum-free medium was 1: 30. After transfection 36 h, the cells were prepared in serum-free medium, added into the chambers and incubated for 16 h at 37°C(2–4 × 10^4^ cells for migration and 4–10 × 10^5^ cells for invasion assay). Five hundred or six hundred microliters medium containing 10% FBS was added into the lower well of each chamber for migration assay or invasion assay respectively.

### Dual luciferase reporter assay

8 × 10^4^ BT-549 cells or HEK293T cells per well were seeded in 24-well plates and incubated at 37°C for 24 h, then co-transfected with 100ng of wide-type 3′ UTR plasmids or mutated MTDH 3′UTR plasmids and 100nM of negative control or miR-630 mimics. Cultured at 37°C for 24 h (HEK293T) or 48 h (BT-549), luciferase activity was measured with the dual luciferase reporter assay system (Promega).

### Western blot

Cell or tissue were lysed using 1× SDS-lysis buffer, then the total protein was separated by SDS PAGE and transferred to nitrocellulose membrane (Axygen, Union City, CA). Immunoblotting was performed with a polyclonal antibody against MTDH (40-6400; Invitrogen, Camarillo, CA). β-actin antibody (1:10000; (CP01; Calbiochem, San Diego, CA) was used as an internal loading control. The membranes were washed and incubated with horseradish peroxidase-conjugated anti-mouse or rabbit IgG for 1 hr at room temperature. The antigen–antibody complexes were visualized using an ECL detection kit (Millipore, Billerica, MA) and the expression levels of these proteins were detected with a high sensitive digital imaging equipment (ImageQuant LAS 4000 mini; GE Healthcare Bio-Sciences AB, Uppsala, Sweden).

### *In vivo* metastasis assays

To ascertain whether miR-630 suppress lung metastasis of breast cancer, MDA-MB-231-D3H2LN cells (Xenogen) were employed and infected with lentiviruses carrying empty vector lenti-NC or expression plasmid lenti-miR-630 to generate the stable transfected cell lines (231-LUC-NC, 231-LUC-miR-630). For *in vivo* pulmonary metastasis assays, 5 × 10^5^ cells were suspended in 200 μl phosphate-buffered saline and injected into the lateral tail vein of 5-week-old female NOD/SIDE mice. Then Firefly Luciferase activity was detected on the same day to confirm the same number of cells injected in both groups of 231-LUC-NC or 231-LUC-miR-630. After two weeks, the lung metastasis burden was monitored weekly by bioluminescence imaging (BLI). Mice were anesthetized each time and given intraperitoneal injection of d-luciferin (150 μg/g body wt prepared in phosphate-buffered saline). After 10–15 mins, bioluminescence images were captured with a charge-coupled device camera (IVIS; Xenogen). Seven weeks later, the mice were killed and lung tissues were isolated and fixed with 4% paraformaldehyde for standard immunohistochemistry (IHC) or histochemistry analysis. The MTDH protein level was assessed using the IHC staining of anti-MTDH antibody for human and the stained slides were scored independently by two investigators according to the value of IRS system.

### Statistical analysis

All the statistical analyses were performed by the statistical package for social science (SPSS) (v. 22) (SPSS Institute). The Pearson's χ^2^ test was used to evaluate the correlation between miR-630 expression and MTDH protein expression. The correlation of relative miR-630 expression levels and MTDH protein expression levels in clinical breast cancer tissues was analyzed using linear correlation and regression. The relevance between the clinical characteristics and miR-630 expression levels was analyzed by Student's *t*-test and Jnockheere-Terpstra test. Multivariate analysis was performed with a linear regression model. Data are presented as mean ± standard error. Error bars indicate standard deviation. Generally, Student's *t*-test was used to evaluate the differences between groups. Differences are considered as significant when the *P* value < 0.05.

## SUPPLEMENTARY FIGURES AND TABLES



## References

[1] Gonzalez-Angulo AM, Morales-Vasquez F, Hortobagyi GN (2007). Overview of resistance to systemic therapy in patients with breast cancer. Adv Exp Med Biol.

[2] Jones SE (2008). Metastatic breast cancer: the treatment challenge. Clin Breast Cancer.

[3] Gupta GP, Massague J (2006). Cancer metastasis: building a framework. Cell.

[4] Steeg PS (2006). Tumor metastasis: mechanistic insights and clinical challenges. Nat Med.

[5] Vanharanta S, Massague J (2013). Origins of metastatic traits. Cancer Cell.

[6] Berx G, van Roy F (2009). Involvement of members of the cadherin superfamily in cancer. Cold Spring Harb Perspect Biol.

[7] Cavallaro U, Christofori G (2004). Cell adhesion and signalling by cadherins and Ig-CAMs in cancer. Nat Rev Cancer.

[8] Bartel DP (2004). MicroRNAs: genomics, biogenesis, mechanism, and function. Cell.

[9] Alvarez-Garcia I, Miska EA (2005). MicroRNA functions in animal development and human disease. Development.

[10] Inui M, Martello G, Piccolo S (2010). MicroRNA control of signal transduction. Nat Rev Mol Cell Biol.

[11] Calin GA, Sevignani C, Dumitru CD, Hyslop T, Noch E, Yendamuri S, Shimizu M, Rattan S, Bullrich F, Negrini M, Croce CM (2004). Human microRNA genes are frequently located at fragile sites and genomic regions involved in cancers. Proceedings of the National Academy of Sciences of the United States of America.

[12] Calin GA, Croce CM (2006). MicroRNA signatures in human cancers. Nat Rev Cancer.

[13] Valastyan S, Reinhardt F, Benaich N, Calogrias D, Szasz AM, Wang ZC, Brock JE, Richardson AL, Weinberg RA (2009). A pleiotropically acting microRNA, miR-31, inhibits breast cancer metastasis. Cell.

[14] Ryu S, McDonnell K, Choi H, Gao D, Hahn M, Joshi N, Park SM, Catena R, Do Y, Brazin J, Vahdat LT, Silver RB, Mittal V (2013). Suppression of miRNA-708 by polycomb group promotes metastases by calcium-induced cell migration. Cancer Cell.

[15] Krichevsky AM, Gabriely G (2009). miR-21: a small multi-faceted RNA. J Cell Mol Med.

[16] Huang Q, Gumireddy K, Schrier M, le Sage C, Nagel R, Nair S, Egan DA, Li A, Huang G, Klein-Szanto AJ, Gimotty PA, Katsaros D, Coukos G, Zhang L, Pure E, Agami R (2008). The microRNAs miR-373 and miR-520c promote tumour invasion and metastasis. Nat Cell Biol.

[17] Park SM, Gaur AB, Lengyel E, Peter ME (2008). The miR-200 family determines the epithelial phenotype of cancer cells by targeting the E-cadherin repressors ZEB1 and ZEB2. Genes Dev.

[18] Gregory PA, Bert AG, Paterson EL, Barry SC, Tsykin A, Farshid G, Vadas MA, Khew-Goodall Y, Goodall GJ (2008). The miR-200 family and miR-205 regulate epithelial to mesenchymal transition by targeting ZEB1 and SIP1. Nat Cell Biol.

[19] Liang YJ, Wang QY, Zhou CX, Yin QQ, He M, Yu XT, Cao DX, Chen GQ, He JR, Zhao Q (2013). MiR-124 targets Slug to regulate epithelial-mesenchymal transition and metastasis of breast cancer. Carcinogenesis.

[20] Kang DC, Su ZZ, Sarkar D, Emdad L, Volsky DJ, Fisher PB (2005). Cloning and characterization of HIV-1-inducible astrocyte elevated gene-1, AEG-1. Gene.

[21] Tokunaga E, Nakashima Y, Yamashita N, Hisamatsu Y, Okada S, Akiyoshi S, Aishima S, Kitao H, Morita M, Maehara Y (2014). Overexpression of metadherin/MTDH is associated with an aggressive phenotype and a poor prognosis in invasive breast cancer. Breast Cancer.

[22] Hu G, Chong RA, Yang Q, Wei Y, Blanco MA, Li F, Reiss M, Au JL, Haffty BG, Kang Y (2009). MTDH activation by 8q22 genomic gain promotes chemoresistance and metastasis of poor-prognosis breast cancer. Cancer Cell.

[23] Brown DM, Ruoslahti E (2004). Metadherin, a cell surface protein in breast tumors that mediates lung metastasis. Cancer Cell.

[24] Emdad L, Lee SG, Su ZZ, Jeon HY, Boukerche H, Sarkar D, Fisher PB (2009). Astrocyte elevated gene-1 (AEG-1) functions as an oncogene and regulates angiogenesis. Proceedings of the National Academy of Sciences of the United States of America.

[25] Zhang B, Liu XX, He JR, Zhou CX, Guo M, He M, Li MF, Chen GQ, Zhao Q (2011). Pathologically decreased miR-26a antagonizes apoptosis and facilitates carcinogenesis by targeting MTDH and EZH2 in breast cancer. Carcinogenesis.

[26] Valastyan S, Weinberg RA (2011). Tumor metastasis: molecular insights and evolving paradigms. Cell.

[27] Benton G, Kleinman HK, George J, Arnaoutova I (2011). Multiple uses of basement membrane-like matrix (BME/Matrigel) in vitro and in vivo with cancer cells. Int J Cancer.

[28] Kleinman HK, Martin GR (2005). Matrigel: basement membrane matrix with biological activity. Semin Cancer Biol.

[29] Friedman RC, Farh KK, Burge CB, Bartel DP (2009). Most mammalian mRNAs are conserved targets of microRNAs. Genome research.

[30] Kikuchi A, Monga SP (2015). PDGFRalpha in liver pathophysiology: emerging roles in development, regeneration, fibrosis, and cancer. Gene expression.

[31] Shen X, Li CC, Aponte AM, Shen RF, Billings EM, Moss J, Vaughan M (2012). Brefeldin A-inhibited ADP-ribosylation factor activator BIG2 regulates cell migration via integrin beta1 cycling and actin remodeling. Proceedings of the National Academy of Sciences of the United States of America.

[32] Emdad L, Das SK, Dasgupta S, Hu B, Sarkar D, Fisher PB (2013). AEG-1/MTDH/LYRIC: signaling pathways, downstream genes, interacting proteins, and regulation of tumor angiogenesis. Adv Cancer Res.

[33] Sobral LM, Sousa LO, Coletta RD, Cabral H, Greene LJ, Tajara EH, Gutkind JS, Curti C, Leopoldino AM (2014). Stable SET knockdown in head and neck squamous cell carcinoma promotes cell invasion and the mesenchymal-like phenotype in vitro, as well as necrosis, cisplatin sensitivity and lymph node metastasis in xenograft tumor models. Molecular cancer.

[34] Fantozzi A, Christofori G (2006). Mouse models of breast cancer metastasis. Breast Cancer Res.

[35] Hanahan D, Weinberg RA (2011). Hallmarks of cancer: the next generation. Cell.

[36] Zhao JJ, Chen PJ, Duan RQ, Li KJ, Wang YZ, Li Y (2014). Up-regulation of miR-630 in clear cell renal cell carcinoma is associated with lower overall survival. International journal of clinical and experimental pathology.

[37] Corcoran C, Rani S, Breslin S, Gogarty M, Ghobrial IM, Crown J, O'Driscoll L (2014). miR-630 targets IGF1R to regulate response to HER-targeting drugs and overall cancer cell progression in HER2 over-expressing breast cancer. Molecular cancer.

[38] Galluzzi L, Morselli E, Vitale I, Kepp O, Senovilla L, Criollo A, Servant N, Paccard C, Hupe P, Robert T, Ripoche H, Lazar V, Harel-Bellan A, Dessen P, Barillot E, Kroemer G (2010). miR-181a and miR-630 regulate cisplatin-induced cancer cell death. Cancer research.

[39] Zhang JW, Li Y, Zeng XC, Zhang T, Fu BS, Yi HM, Zhang Q, Jiang N (2015). miR-630 overexpression in hepatocellular carcinoma tissues is positively correlated with alpha-fetoprotein. Medical science monitor.

[40] Kuo TC, Tan CT, Chang YW, Hong CC, Lee WJ, Chen MW, Jeng YM, Chiou J, Yu P, Chen PS, Wang MY, Hsiao M, Su JL, Kuo ML (2013). Angiopoietin-like protein 1 suppresses SLUG to inhibit cancer cell motility. The Journal of clinical investigation.

[41] Wan L, Lu X, Yuan S, Wei Y, Guo F, Shen M, Yuan M, Chakrabarti R, Hua Y, Smith HA, Blanco MA, Chekmareva M, Wu H, Bronson RT, Haffty BG, Xing Y (2014). MTDH-SND1 interaction is crucial for expansion and activity of tumor-initiating cells in diverse oncogene- and carcinogen-induced mammary tumors. Cancer Cell.

