# Supplementary material for: MiR-630 suppresses breast cancer progression by targeting metadherin
Source: Oncotarget. 2015 Nov 16;7(2):1288–99. doi: 10.18632/oncotarget.6339 (PMC4811460; doi:10.18632/oncotarget.6339)
Supplement: Supplementary file 1 [file oncotarget-07-1288-s001.pdf]

SUPPLEMENTARY FIGURES AND TABLES

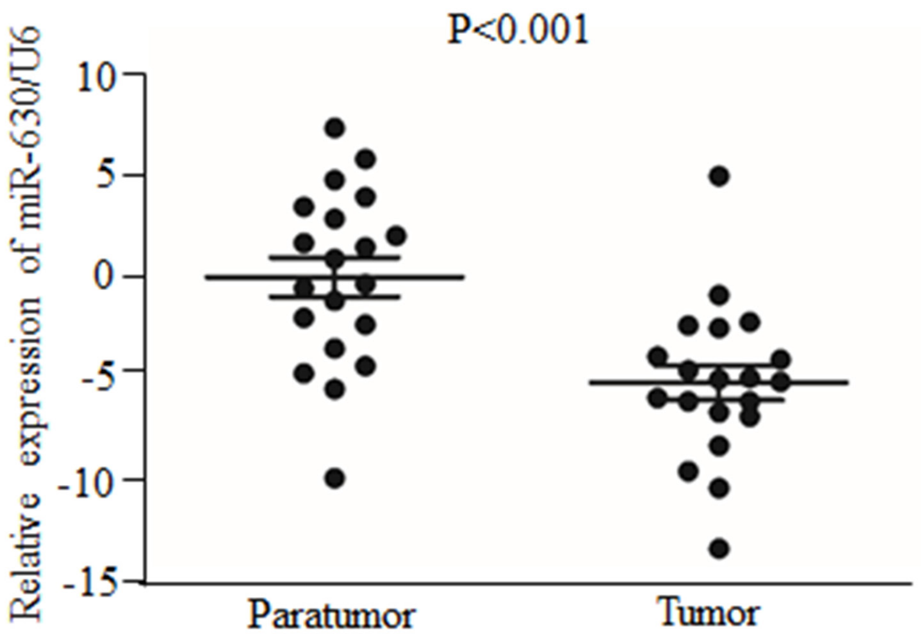

Supplementary Figure S1: MiR-630 is downregulated in breast cancer tissues of validation cohort. Quantitative PCR for comparing the expression levels of miR-630 in 20 paired clinical breast cancer cases. Data represent mean ± SD of three independent experiments. $^{**}P < 0.001$; $^{**}P < 0.001$.

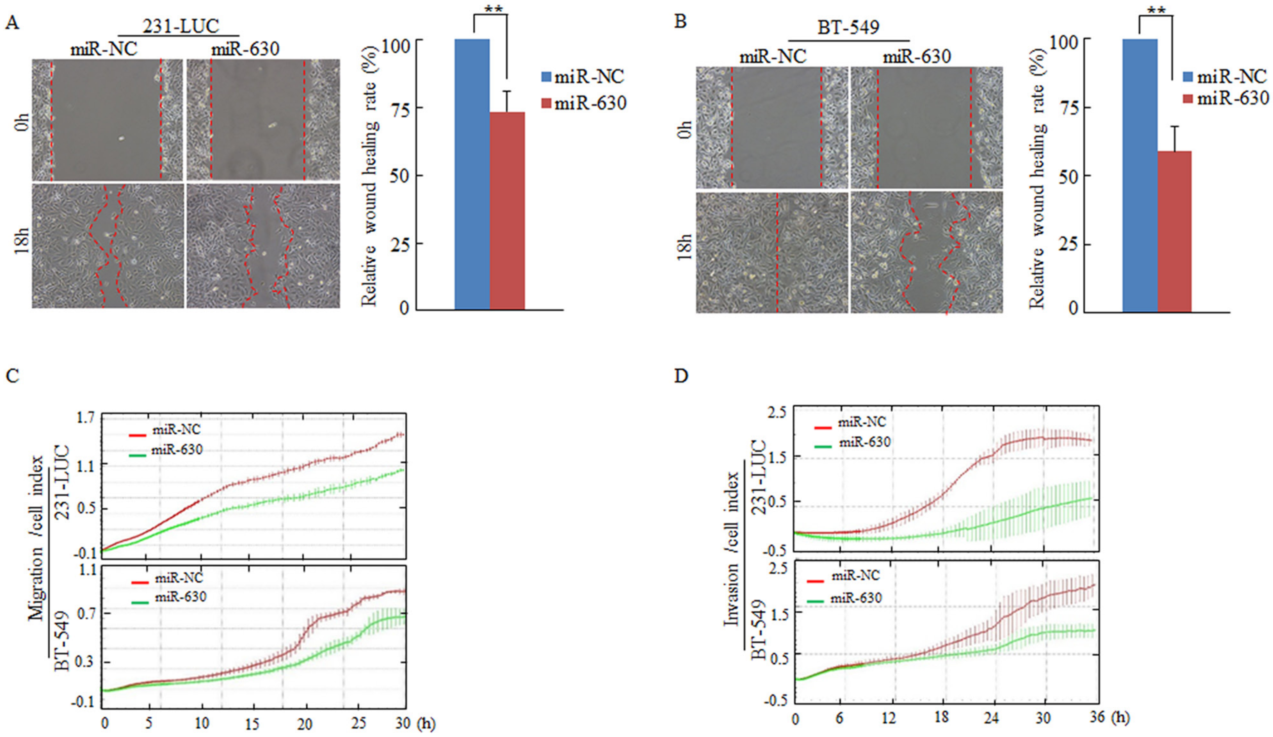

Supplementary Figure S2: miR-630 suppresses breast cancer cell movement *in vitro*. A, B. Wound healing assay measuring cells migration of 231-LUC cells (A) and BT-549 cells (B) transfected with miR-NC or mature miR-630 mimics.36 h after cell tansfection, scratches were created and the images were captured immediately (0 h). Then cells were re-incubated for 18 hours at 37°C and the images were captured again (18 h). Data represent mean ± SD of nine randomly selected areas from three independent experiments, $^{*}P < 0.05$; $^{**}P < 0.01$. **C.** Dynamic monitoring of cell migration using the xCELLigence system. Cells transfected with miR-NC or mature miR-630 mimics were seeded into a CIM-Plate and subjected to a dynamic migration assay lasting for 30 h. **D.** Dynamic monitoring of cell traversing Matrigel using the xCELLigence system. Cells transfected with miR-NC or mature miR-630 mimics were seeded into a CIM-Plate with Matrigel and subjected to a dynamic invasion assay lasting for 36 h.

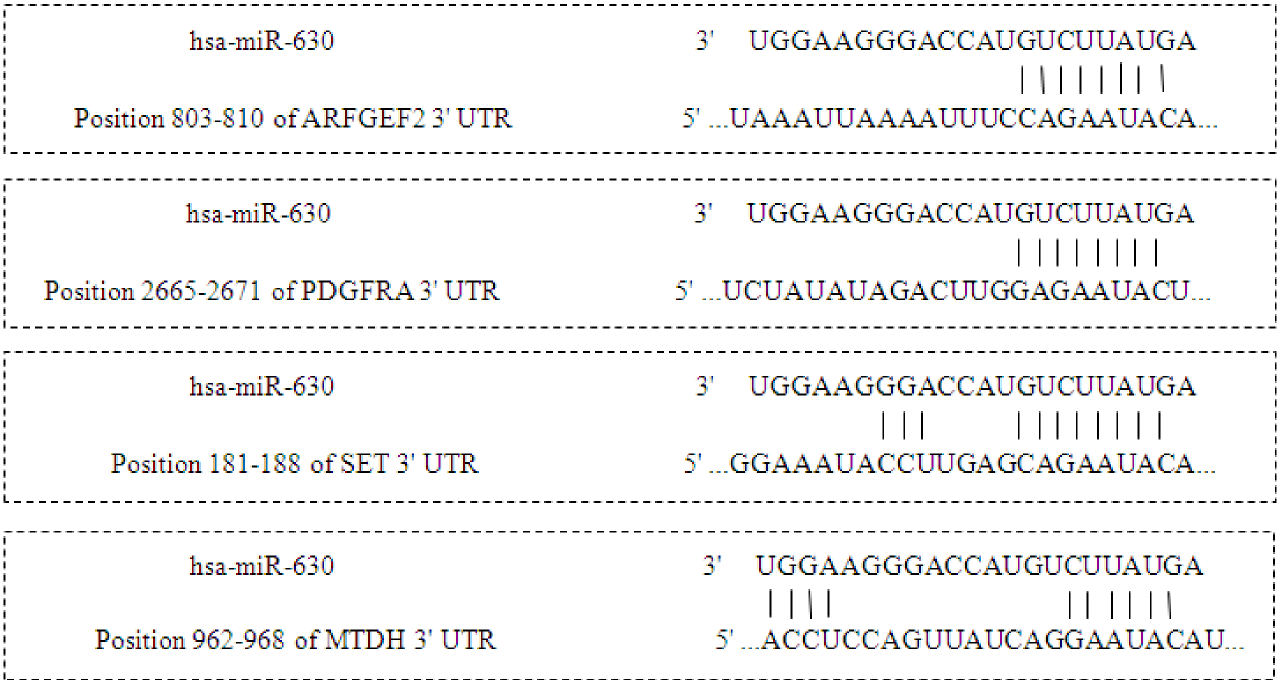

Supplementary Figure S3: Multiple tumor associated genes are predicted as the target genes of miR-630. A. A schematic diagram illustrating the miR-630-binding sites in the potential target genes. **B.** Luciferase reporter assays confirming the potential target genes of the miR-630. All experiments were repeated independent three times.

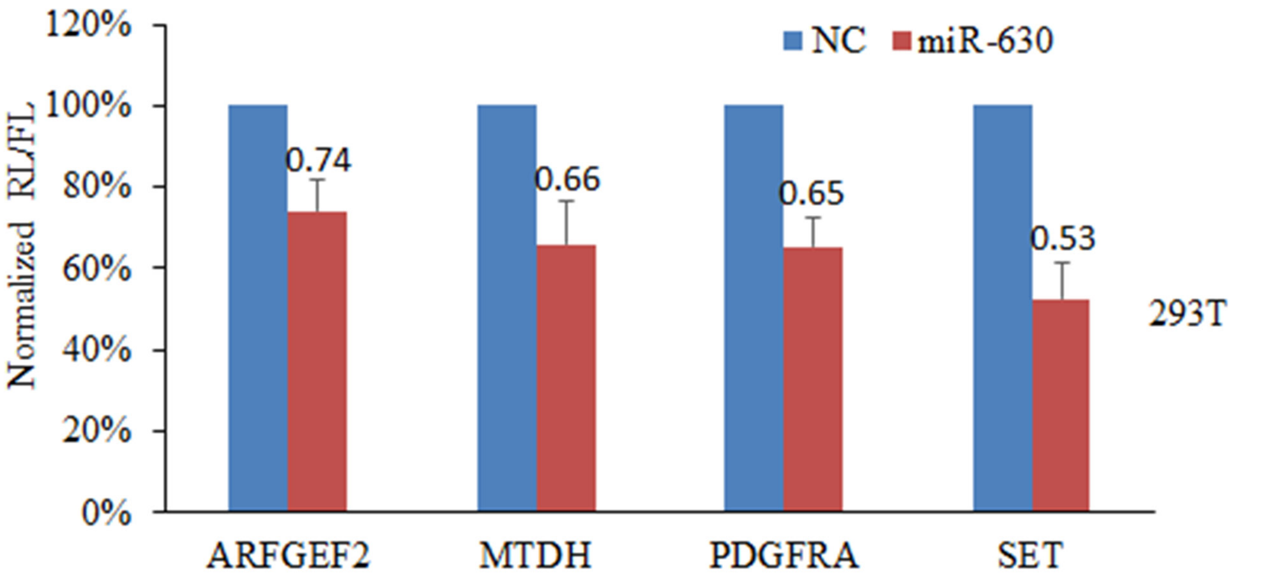

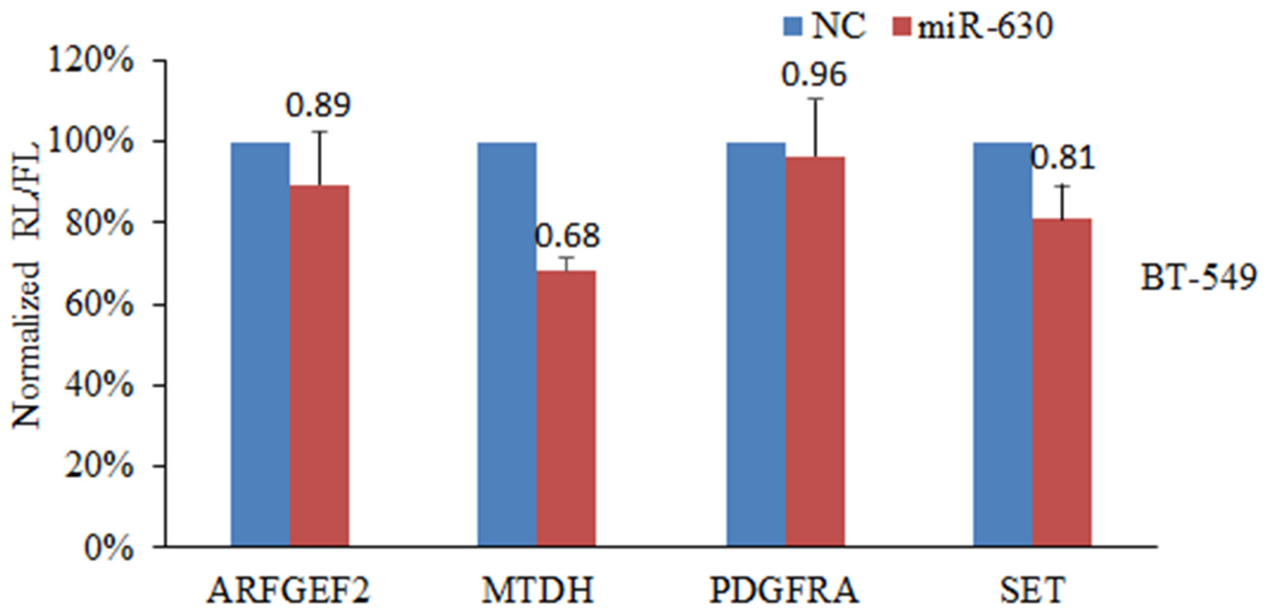

Supplementary Figure S4: Validation of the predicted targets of miR-630. 3′-UTR Luciferase reporter assays of predicted target genes of miR-630 in HEK293T **A.** and BT-549 **B.** cells. Data represent mean ± SD. *$P < 0.05$; **$P < 0.01$; ***$P < 0.001$; All experiments were repeated independent three times.

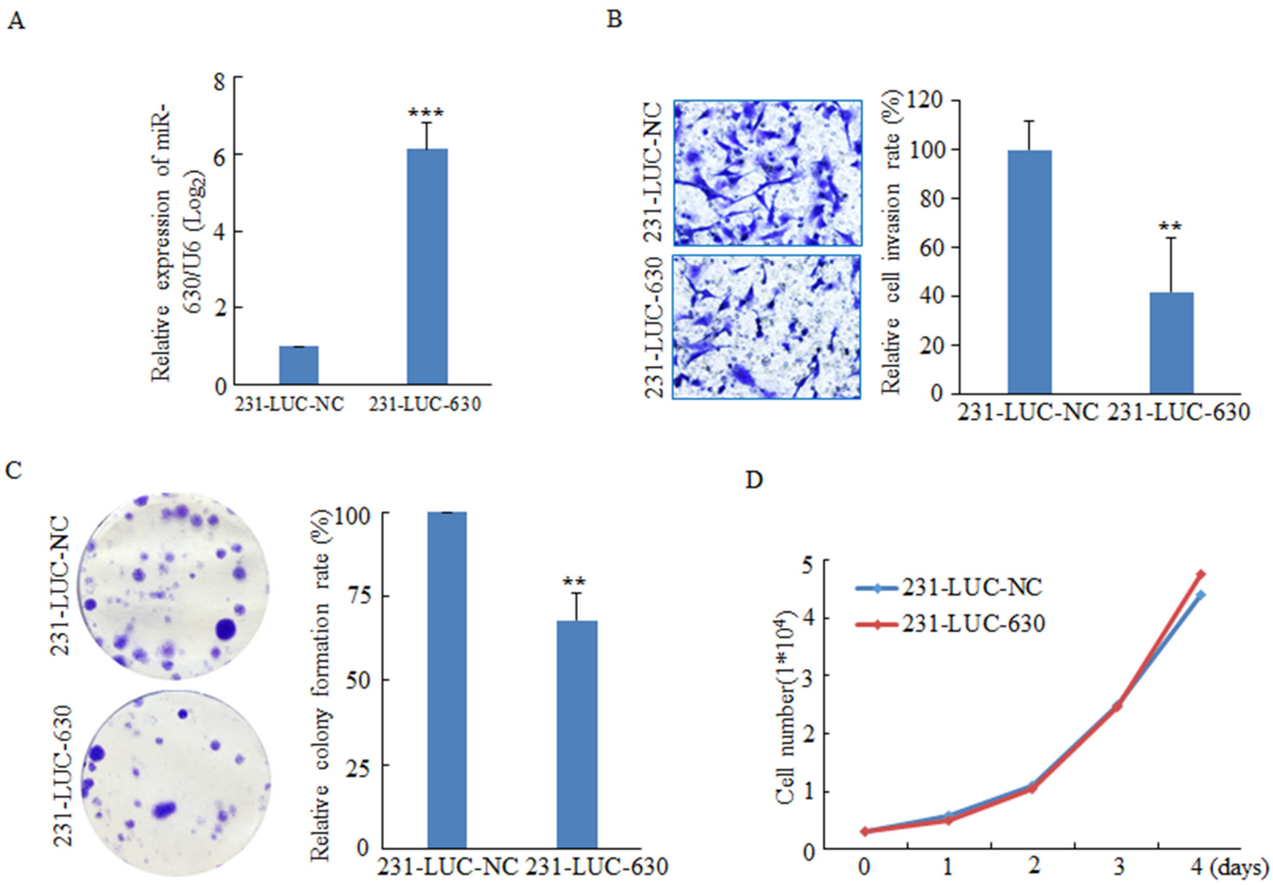

Supplementary Figure S5: Stable overexpression miR-630 impairs breast cancer cells invasion and colony formation A. RT-qPCR showing ectopic expression of miR-630 in 231-LUC cells. B. Effects of stable overexpression of miR-630 on Matrigel invasion of 231-LUC cells. **C.** Effects of stable overexpression of miR-630 on the clonogenic ability of 231-LUC. **D.** The curve depicting for cell proliferation of 231-LUC-NC cells and 231-LUC-miR-630. Cells were embeded into 96-well plates and CCK-8 assay monitored for four days. Data represent mean ± SD from three independent experiments. $^{**}P < 0.01$; $^{***}P < 0.001$. All experiments were repeated independent three times.

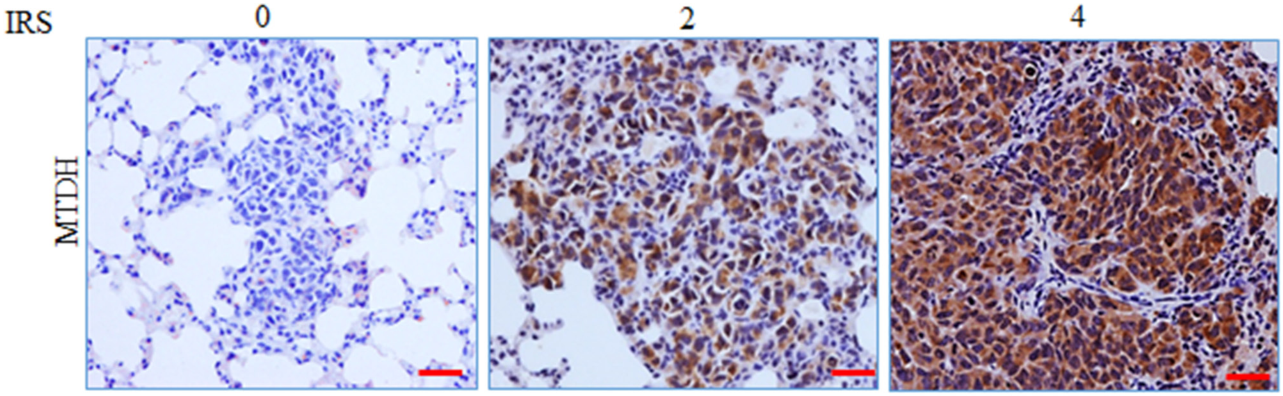

Supplementary Figure S6: Representative images of lung sections from breast cancer metastasis xenografted mice with different degrees of IRS of MTDH expressions based on the IHC staining. The scale bar represents 100 μm.

Supplementary Table S1: Primers for luciferase reporter plasmids and expressing vectors

Name	Sequences
ARFGEF2-UTR-F	5′ AATTCTAGGCGATCGCTCGAGAGCACTGTCACCAGTGTGGTAG
ARFGEF2-UTR- R	5′ ATTTTATTGCGGCCAGCGGCCGCTAAATATTTGATGCAAATATGCAG
PDGFRA-UTR-F	5′ AATTCTAGGCGATCGCTCGAGTGGCGGATTCGAGGGGTTCCTTC
PDGFRA-UTR- R	5′ ATTTTATTGCGGCCAGCGGCCGCGTATTGGTAGACCCTATCAAGTTAG
SET-UTR-F	5′ ATTCTAGGCGATCGCTCGAGATAGAACACTGATGGATTCCAACCTTCCT
SET-UTR- R	5′ TTATTGCGGCCAGCGGCCGCTCTTTATGTTCCTTTATTGGAGCAAGATTC
MTDH-UTR-F	5′ AATTCTAGGCGATCGCTCGAGACACTTGTCTTGAAGATTA-TGCTG
MTDH-UTR- R	5′ ATTTTATTGCGGCCAGCGGCCGCTTCCTAGTCTGACAACGCAATAC
MTDH-UTR-mut-F	5′ CCAGTTATCAGCGTAAAGATTTTTTTACTGCCTTAACCTGTAGTGCGTAGAATATGC
MTDH-UTR-mut-R	5′ GGCAGTAAAAAAATCTTTACGTGATAACTGGAGGTGTTG-ACAATGTCAATTAAAGC
miR-630-F	5′ GGAGGGAGAGGGGCGGGATCCATCAACCACTTTGTA
miR-630-R	5′GGAGGGAGAGGGGCGGGATCCATCAACCACTTTGTA

Supplementary Table S2: Characteristics of clinical patients with breast cancer in the training cohort

Patients no.	Age (year)	Tumor size (cm × cm)	Grade	TNM stage	ER	PR	HER2	Metastasis node count	MIB-1	E-Cadherin	Histological subtype	Location
1	38	3 × 3	3	3	0	0	3	23/26	90%	+	Ductal	Rright
2	57	2.5 × 2.5	3	2	0	0	0	0/17	85%	+	Ductal	Left
3	81	4 × 3.5	3	2	0	0	2	0/16	80/%	+	Ductal	Rright
4	59	2.5 × 2	3	2	0	0	2	0/13	80/%	+	Ductal	Rright
5	69	2.5 × 2	2	1	2	0	3	3/17	80/%	+	Ductal	Left
6	30	6 × 6 × 5	3	4	0	0	1	0/22	80/%	+	Ductal	Rright
7	61	4 × 2	3	2	0	0	2	0/13	70%	+	Ductal	Rright
8	47	4 × 2 × 2	3	2	0	0	3	0/10	70%	+	Ductal	-
9	74	1 × 1	2	1	3	2	1	0/24	70%	+	Ductal	Left
10	38	3 × 2	3	3	3	2	2	22/25	70%	-	Lobular and colloid	Left
11	46	1 × 0.5	3	2	1	1	2	3/21	60%	+	Ductal	Rright
12	56	3.5 × 3	2	2	3	0	3	2/9	60%	+	Ductal	Rright
13	32	5 × 2.5	2	3	0	0	3	2/25	60%	+	Ductal	Rright
14	54	1.5 × 1	2	1	2	0	3	0/20	50%	+	Ductal	Left
15	54	4 × 2.5	3	3	0	0	0	13/39	50%	+	Ductal	Rright
16	63	3 × 2.5	-	2	0	0	2	1/15	5%	+	Apocrine	Left
17	49	4 × 3	2	2	1	0	1	0/21	5%	+	Ductal	Rright

(*Continued*)

Patients no.	Age (year)	Tumor size (cm × cm)	Grade	TNM stage	ER	PR	HER2	Metastasis node count	MIB-1	E-Cadherin	Histolog-ical subtype	Location
18	75	2 × 1.8	2	1	0	0	2	0/16	40%	+	Ductal	Left
19	40	8 × 7 × 4	2	4	1	1	2	20/20	40%	+	Ductal	Rright
20	50	2 × 1.8 × 1.5	3	1	0	0	3	0/9	30%	_	Ductal	Rright
21	37	2.5 × 2 × 2	2	2	3	2	2	3/6	30%	+	Ductal	Rright
22	58	3.5 × 2	3	2	0	0	3	0/14	30%	+	Ductal	Left
23	61	2 × 2	3	3	3	3	3	8/14	20%	+	Ductal	Rright
24	70	4 × 2.5	2	3	3	3	2	17/20	20%	+	Ductal	Rright
25	50	1.5 × 1	2	2	3	0	2	0/13	20%	+	Ductal	Left
26	70	4 × 3	2	2	2	1	1	0/17	10%	+	Ductal	Rright
27	43	3 × 1.5	2	2	2	3	1	1/19	10%	+	Ductal	Rright
28	79	6 × 4.5	2	3	3	3	3	5/13	10%	+	Ductal	Rright
29	56	5 × 2	-	3	1	0	2	14/14	10%	-	Lobular and colloid	Left
30	53	2 × 1.5	2	1	3	3	1	0/14	10%	+	Ductal	Left
31	61	0.7 × 0.7	2	1	3	2	2	8/14	20%	+	Ductal	Rright
32	63	2.8 × 2.5	3	2	3	3	2	17/20	20%	+	Ductal	Rright
33	46	2.5 × 2.5	3	2	0	0	2	0/21	70%	+	Ductal and Medullary	Rright
34	62	1.8 × 0.8	3	2	0	0	2	0/13	60%	+	Ductal	Left
35	-	-	3	2	3	2	1	-	40%	+	Ductal	Rright
36	57	3 × 2.5 × 2.5	3	2	3	1	3	0/16	30%	+	Ductal	Left
37	56	5 × 4	3	2	1	0	2	0/17	30%	+	Medullary	Left
38	37	3 × 2	2	2	1	1	3	2/13	30%	+	Ductal	Rright
39	53	3.5 × 1.5	2	2	3	3	0	0/19	20%	+	Ductal	Left
40	48	2.5 × 2.5	3	2	2	1	3	0/4	15%	+	Ductal	Left
41	50	3 × 3	3	2	0	0	3	-	15%	+	Ductal	Left
42	49	3.5 × 3.5	2	2	3	2	1	1/21	10%	+	Ductal	Left
43	49	-	2	2	3	3	1	-	5%	+	Ductal	-

Note: ER, estrogen receptor; PR, progesterone receptor; For the values of ER, PR and HER2, 3 stands for strongly positive, 1 and 2 stand for mildly positive, 0 stands for negative; - means missing data. + stands for positive of E-Cadherin.

Supplementary Table S3: Characteristics of clinical patients with breast cancer in the validation cohort

Patients no.	Age (year)	Tumor size (cm × cm)	Grade	TNM stage	ER	PR	HER2	Metastasis node count	MIB (%)	Histolog-ical subtype	Location
1	34	1.8 × 1.5	2	1	3	0	2	1/3	-	Ductal	Left
2	78	1.5 × 1.2	3	1	2	1	2	0/17	50%	Ductal	Rright
3	68	3 × 2	3	2	0	0	3	0/16	70/%	Ductal	Rright
4	54	3.5 × 4	2	2	0	1	1	0/13	30/%	Ductal	Rright
5	72	4.5 × 3.5	2	2	0	0	3	2/7	70/%	Ductal	Left
6	29	2.5 × 2	3	2	2	1	1	0/22	80/%	Ductal	Rright
7	81	3 × 2	2	2	2	3	2	6/13	10%	Ductal	Rright
8	53	-	3	1	0	0	3	0/10	-	Ductal	Left
9	48	5 × 4.5	3	1	0	0	3	1/14	70%	Ductal	Rright
10	45	2.5 × 2	2	2	2	-	2	7/25	5%	Ductal	Rright
11	61	2 × 2	3	1	2	0	2	1/11	60%	Ductal	Left
12	35	2.5 × 2	3	2	2	2	3	0/9	40%	Ductal	Rright
13	40	3 × 2.5	1	-	0	0	3	0/10	30%	Ductal	Rright
14	64	2 × 1.5	2	1	0	0	0	1/2	30%	Ductal	Left
15	44	1 × 1.5	-	1	0	0	0	0/10	50%	Ductal	Rright
16	43	1 × 2.5	-	2	1	1	1	1/12	15%	Ductal	Left
17	63	2 × 2.5	2	3	1	0	2	12/34	15%	Ductal	Rright
18	42	2 × 1.8	2	2	1	1	3	0/15	30%	Ductal	Left
19	73	1 × 1.4	2	1	1	0	1	0/5	10%	Ductal	Rright
20	40	2 × 1.5	2	2	0	1	3	0/9	80%	Ductal	Rright

Note: ER, estrogen receptor; PR, progesterone receptor; For the values of ER, PR and HER2, 3 stands for strongly positive, 1 and 2 stand for mildly positive, 0 stands for negative; - means missing data.

Supplementary Table S4: Relevance between the clinical characteristics and miR-630 expression levels

Factors	Patients Number (%)	$\log_2$ (fold repression of miR-630) (mean ± SD)	Univariate analysis	Multivariate analysis
			p-value	*p*-value
**Age (year)**			0.667[a]	0.508[c]
≤50	19 (44.2)	-3.44 ± 4.03		
>50	24 (55.8)	-4.00 ± 4.42		
Missing data	0 (0)			
**Tumor size (cm)**			0.253[a]	0.835[c]
≤2	21 (48.8)	-3.08 ± 3.47		
>2	20 (46.5)	-4.57 ± 4.63		
Missing data	2 (4.7)			
**Nodal status**			0.694[a]	0.945[c]
Positive	18 (41.9)	-4.16 ± 5.09		
Negative	22 (51.2)	-3.61 ± 3.65		
Missing data	3 (7.0)			
**MIB**			0.169[a]	0.905[c]
≤20%	13 (30.2)	-2.42 ± 3.96		
>20%	30 (69.8)	-4.33 ± 4.25		
Missing data	0 (0)			
**Grade**			0.463[a]	0.617[c]
II	20 (46.5)	-3.39 ± 5.11		
III	21 (48.8)	-4.38 ± 3.32		
Missing data	2 (4.7)			
**TNM Stage**			0.717[b]	0.371[c]
I	8 (18.6)	-2.60 ± 3.38		
II	25 (58.1)	-4.28 ± 4.56		
III	8 (18.6)	-2.58 ± 4.20		
IV	2 (4.7)	-6.40 ± 0.42		
Missing data	0 (0)			
**ER status**			0.771[b]	0.200[c]
Strongly positive	16 (37.2)	-4.54 ± 5.26		
Mildly positive	11 (25.6)	-2.31 ± 3.78		
Negative	16 (37.2)	-3.95 ± 3.20		
Missing data	0 (0)			
**PR status**			0.531[b]	0.293[c]
Strongly positive	8 (18.6)	-3.26 ± 4.47		
Mildly positive	12 (27.9)	-3.47 ± 4.38		

(*Continued*)

**Factors**	**Patients Number (%)**	**log_2 (fold repression of miR-630) (mean ± SD)**	**Univariate analysis**	**Multivariate analysis**
			***p*-value**	***p*-value**
Negative	23 (53.5)	-4.07 ± 4.21		
Missing data	0 (0)			
**HER2 status**			0.826[b]	0.937[c]
Strongly positive	14 (32.6)	-3.78 ± 6.04		
Mildly positive	26 (60.5)	-3.80 ± 3.88		
Negative	3 (7.0)	-3.17 ± 4.33		
Missing data	0 (0)			
**E-cadherin**			0.936[a]	0.726[c]
Positive	40 (93.0)	-3.76 ± 4.28		
Negative	3 (7)	-3.56 ± 3.91		
Missing data	0 (0)			

Note: ER, estrogen receptor; PR, progesterone receptor. Data were presented as mean ± standard deviation.
[a]Independent-Samples T test;
[b]Jonckheere-Terpstra test;
[c]Multivariate linear regression analysis.
